# Thermostable DNA Polymerase from a Viral Metagenome Is a Potent RT-PCR Enzyme

**DOI:** 10.1371/journal.pone.0038371

**Published:** 2012-06-04

**Authors:** Michael J. Moser, Robert A. DiFrancesco, Krishne Gowda, Audrey J. Klingele, Darby R. Sugar, Stacy Stocki, David A. Mead, Thomas W. Schoenfeld

**Affiliations:** Lucigen Corporation, Middleton, Wisconsin, United States of America; Saint Louis University, United States of America

## Abstract

Viral metagenomic libraries are a promising but previously untapped source of new reagent enzymes. Deep sequencing and functional screening of viral metagenomic DNA from a near-boiling thermal pool identified clones expressing thermostable DNA polymerase (Pol) activity. Among these, 3173 Pol demonstrated both high thermostability and innate reverse transcriptase (RT) activity. We describe the biochemistry of 3173 Pol and report its use in single-enzyme reverse transcription PCR (RT-PCR). Wild-type 3173 Pol contains a proofreading 3′-5′ exonuclease domain that confers high fidelity in PCR. An easier-to-use exonuclease-deficient derivative was incorporated into a PyroScript RT-PCR master mix and compared to one-enzyme (Tth) and two-enzyme (MMLV RT/Taq) RT-PCR systems for quantitative detection of MS2 RNA, influenza A RNA, and mRNA targets. Specificity and sensitivity of 3173 Pol-based RT-PCR were higher than *Tth* Pol and comparable to three common two-enzyme systems. The performance and simplified set-up make this enzyme a potential alternative for research and molecular diagnostics.

## Introduction

Reverse transcription PCR (RT-PCR) is a powerful analytical and preparative method for detecting, quantifying and analyzing gene expression and RNA viruses. Most RT-PCR protocols rely on two DNA polymerase (Pol) enzymes; a retroviral reverse transcriptase (RT) to copy RNA into cDNA and a thermostable DNA Pol to amplify the target sequence. We describe a unique single-enzyme alternative to the traditional format based on the innate reverse transcriptase activity of the thermostable 3173 Pol, which was recently isolated from a viral metagenomic library [1], [2]. We believe this is the first report of a reagent enzyme produced from a viral metagenomic library, the first viral Pol shown to be fully thermostable *in vitro* and the first single-enzyme RT-PCR protocol with high sensitivity and specificity comparable to two-enzyme systems.

Despite their wide use and general reliability, existing two-enzyme RT-PCR systems have several documented performance problems attributed to deficiencies inherent in retroviral RTs: 1) poor reagent stability, 2) low fidelity, 3) frequent rearrangements during cDNA synthesis, 4) secondary enzymatic activities (i.e. RNase H and strand switching), 5) bias for specific primers and templates, and 6) inhibition of PCR Pol enzymes [3], [4], [5], [6], [7]. These deficiencies are associated with cloning errors, amplification bias, poor concordance between and within testing labs, and target dependent variation in amplification efficiency [8], [9], [10], [11], [12], [13], [14], [15], [16], [17]. The two-enzyme systems require an initial low temperature reverse transcription step that reduces specificity, increases reaction time, and impairs synthesis through complex secondary structures. The limited shelf stability in solution of retroviral RTs has precluded development of complete RT-PCR enzyme premixes popular for standard PCR. Alternative chemistries based on an improved RT-PCR enzyme are a means of addressing these shortcomings.

Numerous thermostable DNA polymerases have been described and commercialized for PCR [18], [19]. All of these fall into one of two groups of high molecular identity and biochemical similarity; bacterial Pol I-type enzymes and archaeal Pol II-type enzymes [20]. Remarkably, no truly thermostable viral replicase-type Pol has ever been described. The pool of useful RTs consists mainly of retroviral Moloney Murine Leukemia Virus (MMLV) RT and its derivatives and Avian Myeloblastosis Virus (AMV) RT. Substantial effort has been devoted to engineering MMLV RT. Truncating the MMLV RT protein to eliminate RNase H activity [21] fortuitously increased thermostability [22]. However, none of the engineered retroviral RTs are thermostable enough for PCR. The alternative bacterial and archaeal Pols also do not fulfill the goal of a facile single-enzyme RT-PCR reagent. *Thermus thermophilus* (*Tth*) Pol I was originally induced to reverse transcribe by inclusion of manganese ions in the reaction buffer [23]. However, *Tth* Pol in the presence of manganese is highly inaccurate and much less sensitive than the two-enzyme systems and therefore not widely used. Notably absent is a thermostable RT suitable for single enzyme RT-PCR that matches or exceeds the performance of two-enzyme systems with regard to fidelity, sensitivity, specificity and low bias.

Viral Pols are interesting alternatives to bacterial or archaeal Pol I and Pol II enzymes, differing significantly in their biologic roles as replicases rather than as short patch repair and lagging strand polymerases [24]. These enzymes possess many important and highly useful biochemical characteristics. Bacteriophage T5, T7 and phi29 DNA polymerases are highly processive enzymes [25], [26], [27] and the latter two are less prone to slippage [28]. T4 bacteriophage DNA polymerase is extremely accurate [29]. While all of these properties make viral Pols very useful as molecular biology reagents, none is suitable for thermocycling based amplification due to limited thermostability. The need for new Pols that combine the practical advantages of viral enzymes with improved thermostability has motivated the metagenomic screens of viral sequences from thermal springs described in this report.

Viral metagenomes are an unexplored source of sequence diversity for the development of new enzymes. A screen of hot spring viral metagenomes identified thousands of open reading frames [2] including many encoding putative thermostable viral Pols. We describe the discovery and biochemical attributes of one of these, 3173 Pol, its inherent RT activity and its incorporation into a single-enzyme PyroScript® 2X RT-PCR Master Mix. The sensitivity, specificity and overall performance of this mix were compared to available one- and two-enzyme systems using a control MS2 RNA bacteriophage template, the clinically-relevant influenza A RNA and commonly used reference mRNA transcripts.

## Materials and Methods

### Discovery and purification of 3173 Pol

Unless indicated otherwise, standard molecular methods were used [30]. Primers and other oligonucleotides (Table 1) were synthesized by IDT (Coralville, IA). Except where noted, the 3173 Pol reaction buffer used throughout was 20 mM Tris-HCl pH 8.8 at 25°C, 10 mM (NH_4_)_2_SO_4_, 10 mM KCl, 2 mM MgSO_4_, 0.1% Triton X-100, and 200 µM of each dNTP (N = A,C,G,T). Construction, sequencing and BLASTx analysis of viral metagenomic libraries have been described [2]. Clones identified by BLASTx analysis as encoding likely *pol* genes were functionally screened to detect expression of thermostable Pol activity. For these screens, viral proteins were constitutively expressed in the original clones by growth to saturation in 2 ml Luria Broth. Cells were pelleted at 2,800 rcf and suspended in 50 mM Tris-HCl pH 7.5,1 mM EDTA, 0.5 mM DTT, 0.1% Triton X-100, 10% (v/v) glycerol. Cells were lysed by sonication and host proteins were denatured by incubation at 70°C for 10 minutes. Soluble proteins were collected from the supernatant after centrifugation at 11,000 rcf for 10 minutes and assayed for DNA Pol activity based on their ability to extend a 5′ fluorescently labeled oligonucleotide primer. The labeled assay primer was annealed at room temperature to the assay template and incubated for 10 minutes at 70°C with 5 µl of each clarified lysate. Primer extension was detected using an ABI 310 Genetic Analyzer (Applied Biosystems, Foster City, CA) in GeneScan mode. For preparative expression, the coding sequence of 3173 Pol was inserted into pET28 and used to transform BL21(DE3) cells according to the manufacturer (EMD Bioscience, San Diego, CA). Pol proteins were expressed, extracted and heat-treated as described for the functional screen and purified using heparin-agarose and Q-sepharose chromatography.

**Table 1:** Primers and Other Oligonucleotides.

Name	DNA Oligonucleotide Sequence 5′ to 3′	Info	Source
Assay primer	* ROX-TGTCTCAGACAGTCAGACTGCTGACAGATGACTTGCA		This report
Assay Template	AACGTGCAAGTCATCTGTCAGCAGTCTGACTGTCTGAGACA		This report
Fid-f	GTCTGAGGCCCTCAGTCCAGTTACGCTGGAGTCTGAGGCTCGT		This report
Fid-r	GAGGGCCTTCATTAGAAAAACTCATCGAGCATCAAGTGAA		This report
M13-f	CGCCAGGGTTTTCCCAGTCACGAC	6333 to 6310 X02513	This report
MS2-77-f	GTCGCGGTAATTGGCGC	632 to 648 NC_001417	[40]
MS2-77-f	GGCCACGTGTTTTGATCGA	708 to 690 NC_001417	[40]
MS2	AGCCAAGCAGCTAGTTACCAAATC	3557 to 3534 NC_001417	This report
MS2	AACTAGCCAAGCAGCTAGTTACCAA	3561 to 3537 NC_001417	This report
MS2	GGGTGGTAACTAGCCAAGCAGCTA	3568 to 3545 NC_001417	This report
MS2-160-r	CCTGCCGGCCACGTGTTTTGATCGA	714 to 690 NC_001417	[40]
MS2-160-f	TTTAGCAGAGGCCAGGTCGACAGCC	555 to 579 NC_001417	This report
#CF560 MS2-160-f	CF560-TTTAGCAGAGGCCAGGTCGACAGCC	555 to 579 NC_001417	This report
MS2-89-f	CCGCTCGTCGCGGTAATTGGCGC	626 to 648 NC_001417	[40]
MS2-124-f	GCTCTAACTCGCGTTCACAGGCTTACAAAGTAACCT	1438 to 1473 NC_001417	[40]
MS2-124-r	ACACCACCAACAGTCTGGGTTGCCAC	1561 to 1536 NC_001417	[40]
MS2-93-f	CCCGCGCTCTGAGAGCGGCTCTATTG	2227 to 2252 NC_001417	[40]
MS2-93-r	GCCTAAATTCATATGACTCGTTATAGCGGACCGCGT	2319 to 2284 NC_001417	[40]
MS2-217-f	GGGCGTCGACCGAAGTCCTGCAAAAG	497 to 522 NC_001417	This report
MS2-218-f	GGCGTCGACCGAAGTCCTGCAAAAGG	498 to 523 NC_001417	This report
MS2-362-f	ACAAGCGAAGTGGGTCATCGTGGGGT	353 to 378 NC_001417	This report
MS2-243-f	GAAGTGCCGCAGAACGTTGCGAACC	472 to 496 NC_001417	This report
MS2-294-f	GCACGCTCCTGCTACAGCCTCTTCC	421 to 445 NC_001417	This report
FluA-f	CCCAGTGAGCGAGGACTGCAGCGTA	230 to 254 V01099	This report
FluA-r	CCCGTTCCCATTAAGGGCATTTTGGACAAAGC	289 to 258 V01099	This report
actin-144-f	CCTGGCACCCAGCACAAT	1041 to 1058 NM_001101	[58]
actin-144-r	GGGCCGGACTCGTCATAC	1184 to 1167 NM_001101	[58]
actin-821-f	GCACCACACCTTCTACAATG	342 to 361 NM_001101	[59]
actin-821-r	TGCTTGCTGATCCACATCTG	1163 to 1144 NM_001101	[59]
GAPDH-f	TGAAGGTCGGAGTCAACGGATTTG	113 to 136 NM_002046	[60]
GAPDH-r	CATGTGGGCCATGAGGTCCACCAC	1095 to 1072 NM_002046	[60]
μglobulin-f	GGCTATCCAGCGTACTCCAAA	117 to 137 NM_004048	[61]
μglobulin-r	CGGCAGGCATACTCATCTTTTT	362 to 341 NM_004048	[61]
cyclophilin-f	CAGACAAGGTCCCAAAGACAG	160 to 180 NM_021130	[62]
cyclophilin-r	TTGCCATCCAACCACTCAGTC	457 to 437 NM_021130	[62]

* ROX = Carboxy-X-rhodamine.

# CF560 = CalFluor 560.

### Biochemical characterization of 3173 Pol

Pol units were determined by a radioactive nucleotide incorporation assay [19] as the amount of enzyme that incorporates 10 nmol of deoxynucleotides per 30 minutes at 70°C. Enzyme dilutions were incubated for 30 minutes at 70°C with reaction buffer supplemented with 10 mg/ml activated calf thymus DNA and 10 mCi/ml [^33^P] dCTP and unit activity was determined based on counts adhering to a DE81 filter (Whatman, Piscataway, NJ). Single-stranded exonuclease activity was determined by incubating the polymerase in standard buffer supplemented with a [^33^P] dCTP radiolabeled PCR product. This substrate was heated to 95°C for 5 minutes and then cooled to 4°C for 10 minutes prior to incubation with 3173 Pol in reaction buffer. Counts due to free nucleotides were measured after precipitation of polynucleotide substrate with 10% trichloroacetic acid (TCA) for 10 minutes on ice.

Site directed mutagenesis was performed using the QuickChange® Site-Directed Mutagenesis Kits (Agilent, Santa Clara, CA). Kinetics and thermal profiles were determined using the radioactive incorporation assay under pseudo-first order conditions of substrate excess [19]. Thermal stability (half-life) was determined by pre-incubating the enzyme in reaction buffer for varying times and measuring the remaining activity by the same assay. Time points were determined in triplicate and decay kinetics were calculated by least squares linear regression of the inverse natural log of the remaining activity at the time points. Standard PCR was performed using cycling conditions described for PyroPhage 3173 DNA Pol by the manufacturer (Lucigen, Middleton, WI). The processivity assay is a modification of published methods [31]. M13f primer (Table 1) was 5′ end labeled with rhodamine. Mix A contained 50 nM primer, 50 nM M13mp18 single strand DNA, and 0.5 nM of Pol in the standard buffer. Mix B contained 0.25 mM each dNTP (N = A,C,G,T) and 0.6 mg/ml activated calf thymus DNA in reaction buffer. An aliquot of Mix A was incubated at room temperature to anneal primer. The reactions were pre-incubated with enzyme at 70°C and an equal volume of Mix B preheated to 70°C was added. Reactions were stopped at 0, 3, 5 and 10 minutes by addition of 50% formamide, 1 mM EDTA. The extension products were resolved on an ABI PRISM 310 instrument using Data Collection Software and peaks were identified and integrated by GeneScan software (Applied Biosystems, Foster City, CA). Processivity was calculated by the following equation:
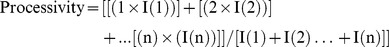
where I = area of each peak, n = number of nt added.

Strand displacement was demonstrated by the ability of 3173 Pol to extend the M13f primer on an M13mp18 ssDNA template for greater than the length of the phage genome (7,249 nt) as determined by 1% agarose gel electrophoresis. Extension from nicks was demonstrated by pre-incubating pUC19 plasmid with Nt.BstNBI nicking enzyme (New England Biolabs, Ipswitch, MA) and incubating the plasmid with 5 units of 3173 Pol for two hours at 55°C. Synthesis was detected by agarose gel.

The Pol fidelity assay was a modification of the *lac*I^q^ reversion assay [32]. The template for this assay was constructed by inserting PCR-amplified *lac*I^q^ coding DNA into the cloning site of pSMART HCKan vector (Lucigen), creating pSMIQ. Primers Fid-f and Fid-r (Table 1) were used to amplify a sequence containing the *lac*I^q^ and *kan* genes. 3173 wild-type and exo- Pols were compared to Pfu (Agilent), Phusion (New England Biolabs) and Taq (Lucigen) Pols. Each of the Pol enzymes was tested according to the respective manufacturer recommendations. The amplicons were digested with Eco0109 I restriction enzyme and ligated to dephosphorylated, Eco0109 I-digested pUC19 vector. The resulting construct was used to transform 10G supreme cells (Lucigen) that were plated on YT agarose plates containing 0.02% (w/v) X-Gal, 0.3 mM IPTG, 100 µg/ml carbenicillin, and 30 µg/l kanamycin. The plates were incubated 20 hours at 37°C and the number of blue and white colonies was determined visually. Fidelity was calculated using the published formula [32]: Fidelity = −lnF/d * t, where F = fraction white colonies, d = number of duplications during PCR (log_2_ of fold amplification) and t is the effective target size (t = 349 for *lac*I^q^).

The fluorogenic RT assay was performed by incubating 500 ng/µl polyA (Sigma-Aldrich, St. Louis, MO) with 25 ng/µl oligo-dT (Invitrogen, Carlsbad, CA) in 25 µl manufacturer recommended buffer containing 250 nM dTTP using the iCycler MyiQ qPCR instrument (BioRad, Hercules, CA). The 3173 Pol and Taq reactions contained 5 Pol units, the AMV RT (Promega, Madison, WI) reaction contained 10 units and the MMLV RT (New England Biolabs) reaction contained 200 units based on unit definitions of the suppliers. Reactions lacking dTTP were preincubated at 37°C to equilibrate secondary structures of the substrate and reduce high initial fluorescence background. Next 37°C dTTP was added to start the reaction. One hundred fluorescence reads were performed every six seconds at 37°C followed by an additional one hundred fluorescence reads at 65°C. Direct incubation at 65°C does not detect RT activity because the reaction temperature is greater than the melting temperature of the oligo dT primers on the polyA template. Data analysis was performed by linear least squares regression of a plot of fluorescence data in RFU versus reaction time in seconds using data from 30 to 150 seconds of incubation at 37°C and data from 30 to 90 seconds of incubation at 65°C.

RT primer extension assays were performed using the same conditions as the fluorogenic RT assay. Reactions with polyA template employed hexachlorofluorescein (HEX) labeled dT_20_ oligonucleotide instead of oligo dT primer. The RT primer extension assay reactions with MS2 RNA template and CalFluor560-labeled (Biosearch Technologies, Novato, CA) MS2-specific primer (MS2 160-r, Table 2) were incubated for 10 minutes at 37°C, and then 30 minutes at 65°C. For PAGE analysis, reactions were stopped by incubation for 5 minutes at 95°C in 1M urea and held on ice prior to electrophoresis on denaturing 5 or 10% polyacrylamide 1X TBE gels (BioRad). HEX and CalFluor560 fluorescence was detected by a Pharos FX fluorescence scanner (BioRad).

**Table 2:** RT-PCR conditions.

Kit	ReverseTranscription		Denature at 94°C	PCRDenature at 94°C	PCRAnneal		PCRExtension		Finishing	RunTime^a^
	Temp.	Time	Time	Time	Temp.	Time	Temp.	Time		
PyroScript	None	NA	2 min	15 sec	NA		72°C	30 sec	NA	58 min
Quanta	50°C	5 min	2 min	15 sec	NA		72°C	30 sec	NA	63 min
Transcriptor	50°C	30 min	7 min	10 sec	NA		68°C	30 sec	68°C 7 min	100 min
Superscript	55°C	30 min	2 min	15 sec	66°C	30 sec	68°C	30 sec	68°C 5 min	119 min

a Not including thermal melt. NA is not applicable.

### RT-PCR

MS2 RNA bacteriophage (Accession Number NC_001417) was cultivated using published procedures [33]. The MS2 phage particles were precipitated from 0.5 M NaCl and 10% PEG-8000, purified by isopycnic centrifugation in 1.40 g/ml CsCl and dialyzed into 10 mM Tris-HCl pH 7.4, 100 mM NaCl, 0.1 mM MgSO_4_. Phage preparations were adjusted to 50% glycerol and stored frozen. RNA was isolated from thawed aliquots with either the QIAamp MinElute Virus Spin Kit (QIAGEN, Valencia, CA) or the Tri Reagent LS reagent (Molecular Research Center, Inc., Cincinnati, OH) according to manufacturer instructions. Influenza A RNA was isolated from cultures of MCDK cells infected with Influenza A strain A/Puerto Rico/8/1934 (H1N1). Infected cells were clarified by centrifugation and RNA isolated by QIAamp MinElute Virus Spin Kit was frozen immediately. No DNase treatment was used for either preparation. For detection of transcripts, total human liver RNA (Ambion, Austin, TX) was used. For quantification, MS2 RNA was re-suspended in 100 mM EDTA and the RNA concentration was estimated by absorbance at 260 nm with an extinction coefficient of 40 µg ml−1 OD−1. The estimated MS2 RNA copy number was calculated from the determined concentration using an average molecular weight for an RNA base of 340 g mole−1 and the MS2 genome length of 3,569 nt.

Two-step RT-PCR reactions were performed using either 5 units 3173 Pol, exonuclease negative mutant or 200 Units MMLV RT (NEB). RNA was combined with primers and annealed in water at 70°C for 5 minutes followed by incubation on ice. Primers used were oligo dT 12–18 mer, random hexamers, random nonamers, and gene specific primers. No primer and no RT controls were performed. First strand synthesis was performed in manufacturer recommended buffer with 0.5 mM dNTPs for 5 minutes at 25°C and then for 30 minutes at 37°C for the oligo dT, random hexamer, random nonamer and control reactions. Gene specific RT reactions were incubated for 30 minutes at 42°C for MMLV and 60°C for 3173 Pol. Reactions were terminated by incubation at 95°C for 5 minutes. Following reverse transcription a tenth of the reaction was PCR amplified by Taq Polymerase (Lucigen) in 40 cycles of PCR.

For one-step RT-PCR reactions the following conditions were used. The PyroScript® RT-PCR 2X Master Mix (Lucigen) containing 2.5 units of 3173 Pol, was used in reactions at 1X concentration with primers at 200 µM each. The SuperScript® III One-Step RT-PCR System with Platinum® Taq DNA Polymerase (Life Technologies, Carlsbad, CA), the qScript™ One-Step SYBR® Green qRT-PCR Kit (Quanta Biosciences, Gaithersburg, MD), the Transcriptor® One-Step RT-PCR Kit (Roche Applied Science, Mannheim, Germany) and *Tth* DNA polymerase (Epicentre Technologies, Madison, WI) were used according to manufacturer instructions. PCR and real-time PCR were performed using an iCycler® MyiQ™ thermal cycler (BioRad) on sample sizes of 25 µl employing the cycling conditions specified by the respective RT-PCR kit manufacturers (Table 2). For qPCR, amplification data was acquired during the PCR extension step, a thermal melt was performed from 70–95°C. For the Roche and the Lucigen reagents, a fluorescent DNA-binding dye, EvaGreen (Biotium, Hayward, CA), was added at 0.5×. Data acquisition used the iQ Optical System software version 2.1 (Bio-Rad) and analysis was performed using MultiCode-RTx Analysis software version 1.6.2.10 (EraGen Biosciences, Madison, WI).

## Results

### Discovery and expression of 3173 Pol

**Figure 1:**
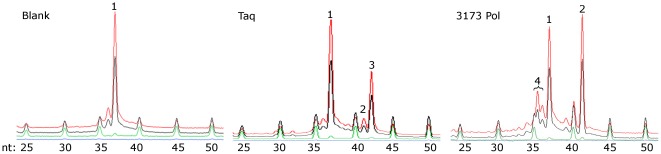
Polymerase assay for detection of expression clones containing thermostable Pol activity from a boiling hot spring metagenomic library. Clones judged by sequence to encode complete *pol* genes were cultivated and thermostable proteins extracted as described in the Methods. Extension of a 37 nucleotide (nt) ROX-labeled primer **(peak 1)** on a 41 nt template oligonucleotide by a polymerase results in a shift from 37 to 41 nt **(peak 2)**. If a single nucleotide non-templated extension occurs as seen with Taq Pol, a peak at 42 nt results **(peak 3)**. Degradation of the ROX-labeled substrate by 3′ to 5′ exonuclease activity results in peaks of less than 37 nt **(peak 4)**. nt: size of standard markers in nucleotides.

A viral metagenomic library was constructed from Octopus hot spring (93°C) in Yellowstone National Park and 21,198 Sanger sequence reads were analyzed [2]. BLASTx alignment [34] to the Genbank protein sequence database identified hundreds of potential *pol* genes. Analysis of paired end reads of individual metagenomic clones suggested 59 complete *pol* genes. All of these were tested for expression of Pol activity using a primer extension assay, and ten clones displayed detectable thermostable Pol activity. The most thermostable of these activities was from clone number 3173, encoding 3173 Pol (Figure 1). This enzyme belongs to a family of thermostable viral Pols identified in this and other screens that have strongest sequence similarity to Pol I-type enzymes from the *Aquificales* family. The 3173 Pol (Genbank acc. no. ADL99605.1) shares 32% amino acid identity with *Thermocrinis albus* Pol I (Genbank acc. no. ADC89878.1), but no significant sequence similarity to any previously described viral protein.

### Biochemical Analysis

The 3173 Pol was over-expressed in *E. coli* and purified. Its biochemical attributes are summarized in Table 3. Protein sequence alignment identified a Pol domain and a 3′-5′ exonuclease domain, but no detectable 5′-3′ exonuclease domain. The primer extension Pol assay also detected 3′-5′ exonuclease activity in the purified Pol preparation (Figure 1) and this activity was further confirmed by digestion and release of acid soluble counts from a radiolabeled DNA fragment (Table S1). The identification of a proofreading exonuclease domain suggested high fidelity synthesis. A variant of the *lac*I^q^ forward mutation fidelity assay [35] was used to determine the fidelity of 3173 Pol in PCR amplification of a DNA target (Table 3). The wild-type 3173 Pol had a fidelity of 6.7×10^4^.

**Table 3:** Biochemical attributes of PyroPhage 3173 Pol.

3′-5′ exonuclease	Strong
5′-3′ exonuclease	None
Strand displacement	Strong
Extension from nicks	Strong
Thermostability (T_½_ @94°)	11.1+/−1.4 min.
K_m_ dNTPs	40 mM
K_m_ DNA	5.3 nM
Processivity	47 nt
3′ ends of amplicons	blunt (wt)single nt extended (exo-)
Fidelity	8×10^4^ (wt)0.9×10^4^ (exo-)

Proofreading exonuclease activity can complicate PCR by degrading unmodified primers and templates [36]. Since fidelity of incorporation is less important for detection and quantification, the exonuclease activity of the 3173 Pol was eliminated to create a more robust enzyme for routine RT-PCR. Sequence alignment to the 3′-5′ exonuclease domains of known Pols [37] predicted that aspartate 49 and glutamate 51 of 3173 Pol would be required for exonuclease activity. Substitution of either acidic residue with alanine eliminated measurable exonuclease activity. As would be expected, disabling the proofreading exonuclease reduced PCR fidelity to 0.9×10^4^. The D49A mutant of 3173 Pol (PyroPhage 3173 DNA Polymerase, Exonuclease Minus, Lucigen) was used for all of the remaining work.

**Figure 2:**
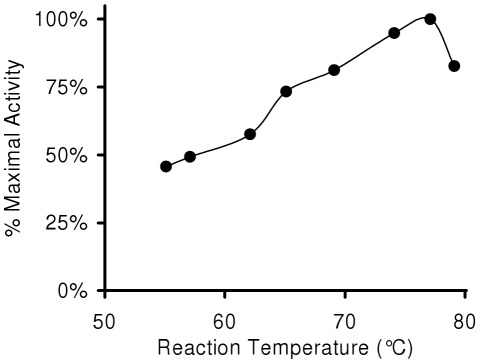
Biochemical characterization of 3173 Pol. The thermal profile of the 3173 Pol was determined by assay at the indicated temperatures. Activity relative to maximal (77°C) is shown.

We determined the processivity of the 3173 enzyme using a variant of the “enzyme trap” method [38], in which Pol was preloaded onto a fluorophore-labeled primer/template complex. Excess activated calf thymus DNA was added simultaneously with nucleotides to capture the Pol in non-detected extension products after enzyme dissociation from the primer/template. Primer extension has traditionally been detected by polyacrylamide gel electrophoresis. Capillary electrophoresis of fluorescently tagged primer extension products allows quantitative determination of processivity by direct measurement of the number of nucleotides incorporated and the amount of each extension product based on electrophoretic mobility and peak integration. Reactions where the observed lengths of extension no longer increased with time were chosen for analysis. This approach showed a mean processivity of 47 nucleotides for 3173 Pol. The same analysis indicated processivity of 9 and 37 nucleotides for *Taq* and *Bacillus stearothermophilus* (*Bst*) Pol I enzymes, respectively. The 3173 enzyme has a half-life at 94°C of about eleven minutes. In contrast Taq Pol measured under the same conditions lost 45% activity over the two hour assay. The thermal profile of 3173 Pol (Figure 2) shows peak activity at 77°C, with approximately half maximal activity at 55°C.

**Figure 3:**
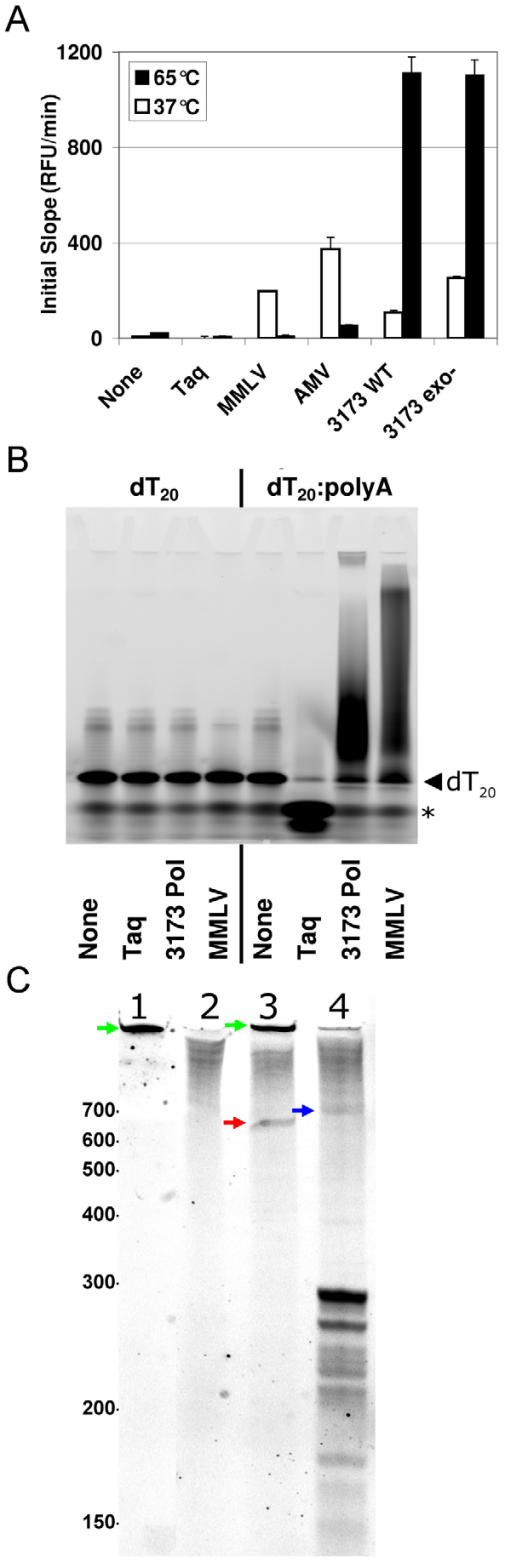
Reverse transcriptase assays. **A.** Fluorogenic assay. RT activity was measured by detection of RNA:DNA heteroduplex by fluorescence of EvaGreen binding. Oligo dT primed poly A was incubated at 37°C and 65°C in the presence of indicated Pol enzymes in manufacturer recommended buffers and dTTP. Fluorescence measurements were obtained every 6 seconds for 10 minutes. The initial slopes from a plot of RFU vs. time in seconds were determined by linear least square regression from 30 to 150 seconds at 37°C and from 30 to 90 seconds at 65°C. Error bars are standard error of regression slope. **B.** RT primer extension assay. HEX-labeled dT_20_ primed poly A was incubated 10 minutes at 37°C and then 10 minutes at 65°C in the presence of indicated Pol enzymes and dTTP in manufacturer recommended buffers. Primer extension products were resolved by 10% denaturing PAGE and imaged on a Molecular Imager FX (Bio-Rad). Left facing triangle indicates migration of unextended dT20 primer and asterisk indicates bromophenol blue dye front. **C.** RT MS2-specific primer extension. 5′-labeled primer was annealed to MS2 RNA and incubated 10 minutes at 37°C and then 30 minutes at 65°C in the presence of indicated Pol enzymes with dNTPS (N = A,C,G,T) in manufacturer recommended buffers. Primer extension products were resolved by 5% denaturing PAGE. Lane 1 No RNA+MMLV RT; Lane 2: MS2 RNA No RT; Lane 3 MS2 RNA+MMLV RT, Lane 3 MS2 RNA+3173 Pol. Molecular weight in bases indicted. Red Arrow: ∼650 base MMLV extension product. Blue Arrow: ∼715 base PyroScript extension product. Green arrow: Non-templated MMLV reaction product.

Both radioactive and fluorogenic incorporation assays indicated strong RNA-dependent DNA synthesis (reverse transcription) activity for 3173 Pol in buffers containing either magnesium or manganese (not shown). We used two assays (Figure 3) to compare the RT activities of the wild-type and exonuclease deficient 3173 Pols to those of AMV and MMLV RTs at 37°C or 65°C on an oligo dT primed poly A substrate. The AMV and MMLV had higher RT activity at 37°C while the 3173 Pol RT was much more active at 65°C using the fluorogenic incorporation assay (Figure 3A). The Taq polymerase and no enzyme controls had no detectable RT activity at either temperature. Extension products from a 5′-fluorophore-labeled dT20 primer were resolved by denaturing polyacrylamide gel to further demonstrate RT activity and to assess the relative lengths of the extension products of the 3173 Pol and MMLV RT (Figure 3B). Both RTs were able to efficiently extend the primer when polyA RNA template was provided. The length distribution of the 3173 Pol cDNAs was visibly shorter than that produced by the MMLV RT, although a subset of the 3173 extension products appeared to be so large that they barely entered the gel. Incubation of the DNA primer:RNA template complex with the Taq Pol negative control resulted in a structure-dependent 5′-3′ exonuclease cleavage product that migrated at the dye front [39]. As an additional test to compare the RT activity of the 3173 Pol to that of MMLV-RT on a complex RNA substrate, a primer specific to bases 714 to 690 of the negative sense RNA MS2 genome [40] was 5′-fluorophore-labeled. The labeled cDNA primer was extended using extracted MS2 RNA as a template (Figure 3C). The 3173 Pol and MMLV RT were both able to extend the primer to produce faint, nearly full-length products although the 3173 Pol product was detectably longer than that of MMLV RT. The 3173 Pol also synthesized a larger amount of several shorter length extension products from 175 to 300 bases in length. The MMLV RT formed a visible template-independent product in the absence of added RNA template that was not resolved by the gel, while the 3173 Pol did not.

### Use of 3173 Pol in RT-PCR

**Figure 4:**
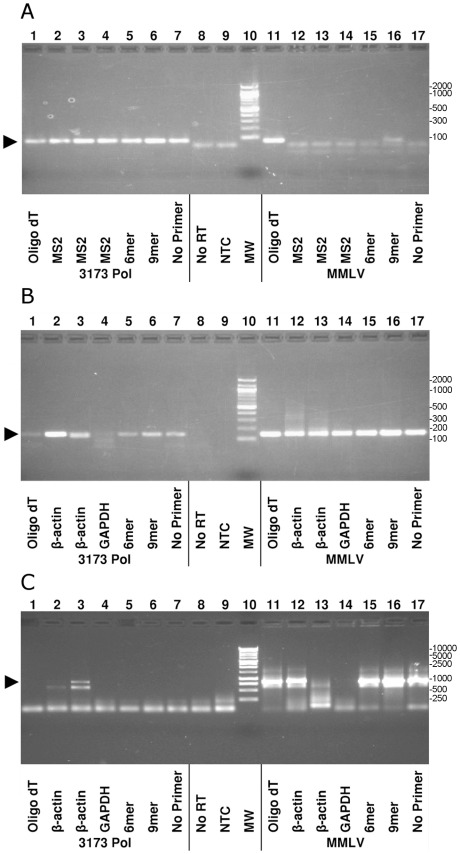
Two step RT-PCR comparing 3173 Pol and MMLV RT. **A.** MS2 viral RNA and **B., C.** total human liver RNA were reverse transcribed using either 3173 Pol or MMLV RT and then PCR amplified using Taq Polymerase. **Target amplicons**: **A.** MS2 RNA phage 77 bp amplicon, 2% gel, **B.** Human beta-actin 144 bp amplicon, 2% gel, **C.** Human beta-actin 821 bp amplicon, 1% gel. **Lanes**: **1**,**11**: oligo dT primer; **2–4**,**12–14**: Gene specific primers; **5**,**15**: random hexamers; **6**,**16**: random nonamers; **7**,**17**: No primer plus RT; **8**: No RT enzyme; **9**: PCR No Target Control; **10**: Molecular Weight Marker (MW), 100 bp (50 bp lowest) for Panels A, B and 1000 bp (300, 500, 700 lowest) for Panel C. Correct PCR product size indicated by black triangle.

We compared the first strand cDNA synthesis by 3173 Pol to that by MMLV RT using biological RNA templates. Production of cDNA was detected by two-step PCR amplification in which cDNA synthesis was primed by random, target-specific, oligo dT or no primers and detected by PCR with target-specific primers (Figure 4). The RNA targets were MS2 bacteriophage and a human mRNA. The 3173 Pol readily synthesized 77 bp MS2 [40] (Lanes A1–7) and 144 bp beta-actin cDNAs (Lane B2, 3, 5, 6, 7) but generally failed to synthesize cDNA targets longer than ∼400 bp (Panel C). Of the two longer target sequences tested, only the 821 bp beta-actin sequence (Lane C3) was reverse transcribed by the 3173 Pol and this synthesis appeared less efficient than that of MMLV RT. Interestingly, both enzymes appeared to reverse transcribe with primers that would not be expected to prime near the target. (A1, A11, B14) and even in the absence of primers (Lane A7, B17, C17). In the case of 3173 Pol, it is likely that both cDNA synthesis and amplification occur during the PCR step so the presence or absence of primers during cDNA synthesis may be inconsequential. The basis of the product in the MMLV RT reaction is not known.

**Figure 5:**
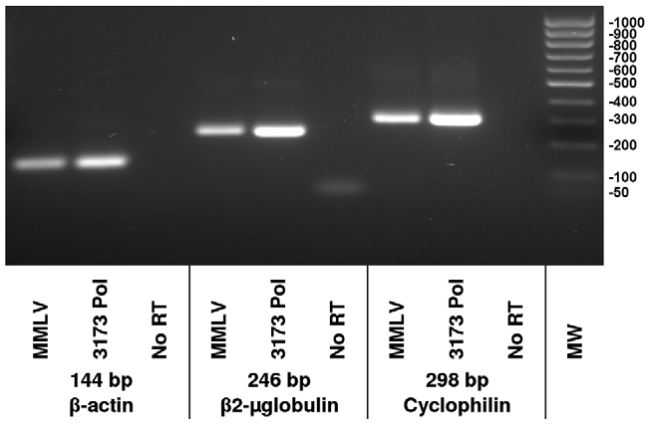
RT-PCR detection of human transcript RNAs. Beta-actin, beta2-microglobulin and cyclophilin target sequences of the indicated sizes were amplified from human liver total RNA using the primers described in Table 1. Shown are products of two step reactions where either MMLV RT or 3173 Pol were used for first strand cDNA synthesis, as indicated. Taq Pol was used for PCR. Products were resolved on a 1% agarose gel.

Under favorable conditions 3173 Pol did reverse transcribe mRNA transcripts (Figure 5). The 3173 Pol was compared to MMLV RT for the detection of three shorter target sequences in common high-abundance reference genes using the two-step RT-PCR protocol. Both enzymes appear to transcribe the targets with similar efficiency and specificity. The amount of PCR product for all three transcripts appeared visibly greater in the 3173 Pol reactions, although we cannot rule out the contribution of residual thermostable 3173 Pol to the PCR reaction yield.

To facilitate RT-PCR, the exonuclease deficient 3173 Pol was combined with buffer and deoxynucleotides to formulate PyroScript® RT-PCR 2X Master Mix (Lucigen) for single-enzyme, one step RT-PCR. Preliminary testing indicated that an initial lower temperature RT extension prior to thermal cycling did not improve results with the PyroScript enzyme (not shown). Therefore this step was eliminated from PyroScript RT-PCR protocols. In contrast to the typical RT-PCR primers designed for the lower extension temperatures of MMLV or AMV RTs, primers used with melting temperatures of about 72°C significantly improved RT-PCR performance of the PyroScript enzyme mix.

**Figure 6:**
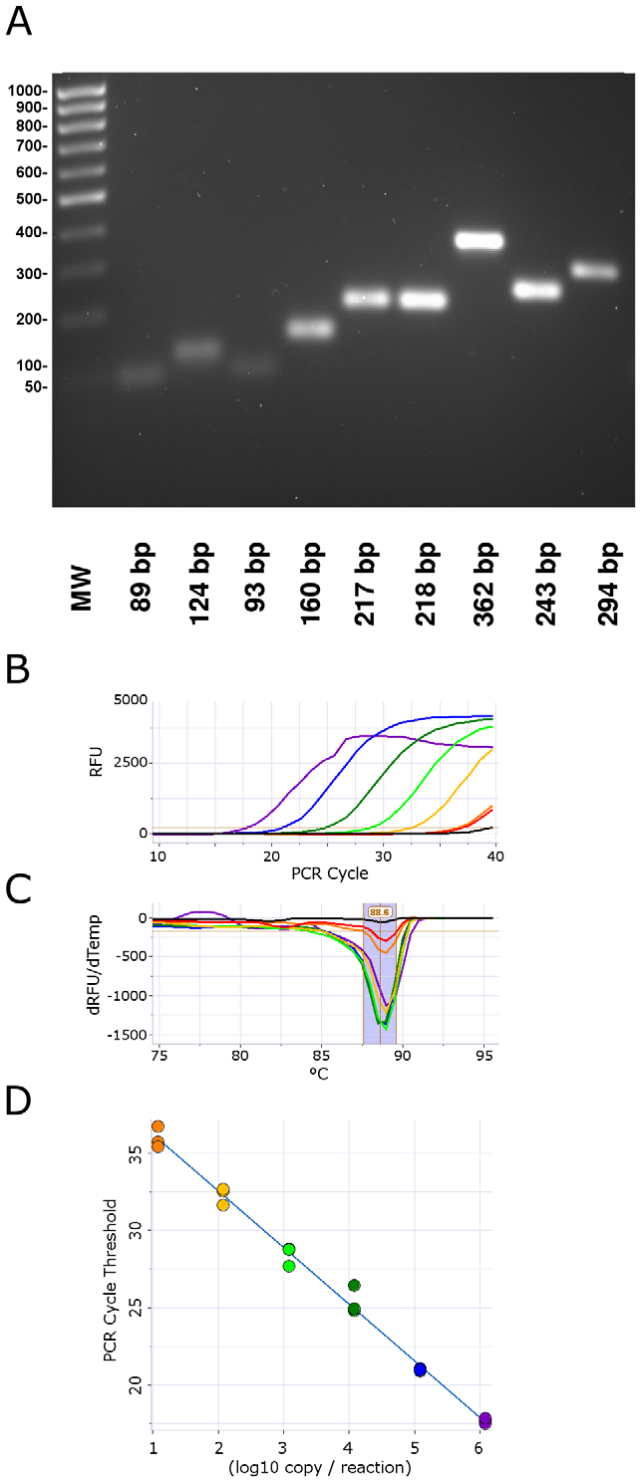
Single-enzyme, one step RT-PCR amplification of MS2 phage RNA using 3173 Pol. MS2 RNA was amplified by 40 cycles of RT-PCR using the primers shown in Table 1 and 3173 Pol. **A.** Products from 89 to 362 bp in length were amplified using one-step single-enzyme RT-PCR cycling conditions: 15 sec @ 94°C, (10 s @ 94°C, 30 s @ 72°C)*40. Products were resolved by 2% agarose gel electrophoresis. **B.** The MS2 RNA was diluted from 10^1^ to 10^7^-fold and amplified using a primer pair corresponding to the 160 bp fragment in Panel A. Real-time PCR fluorescence in RFU (relative fluorescence units) vs. PCR cycles. **C.** Post-amplification thermal melt in -dRFU/dTemperature vs. Temperature (°C). Light blue region indicates melt curves for specific products. **D.** Standard curve PCR Cycle threshold vs. log_10_ RNA copy number in triplicate with linear least squares best fit line.

To assess sensitivity and specificity of the PyroScript master mix reagent in one step RT-PCR, a quantitated control target was prepared from RNA bacteriophage MS2 [41]. We used the one-enzyme PyroScript RT-PCR mix with nine primer sets (Table 1) to amplify regions of the MS2 RNA genome [40] up to 362 bp. The mix proved effective for these primer sets and this range of target sizes (Figure 6A). Amplification efficiency for longer target lengths was poor as judged by a substantial increase in qPCR cycle threshold with amplicons greater than about 400 bp (not shown). To demonstrate quantitation and sensitivity, the 160 bp MS2 primer set from Figure 6A was chosen as well suited for both qPCR and electrophoresis analysis and combined with PyroScript to amplify a ten-fold dilution series from 1,200,000 to 1.2 target copies of MS2 RNA (Figure 6B). The estimated limit of detection was between one and ten RNA copies. The water-only control gave a negative response demonstrating high specificity, which is supported by the melt-curve analysis (Figure 6C) and agarose gel electrophoresis of the product (not shown). Linear quantitation was seen over the full six-log dilution series (Figure 6D) suggesting a broad quantitation range.

**Figure 7:**
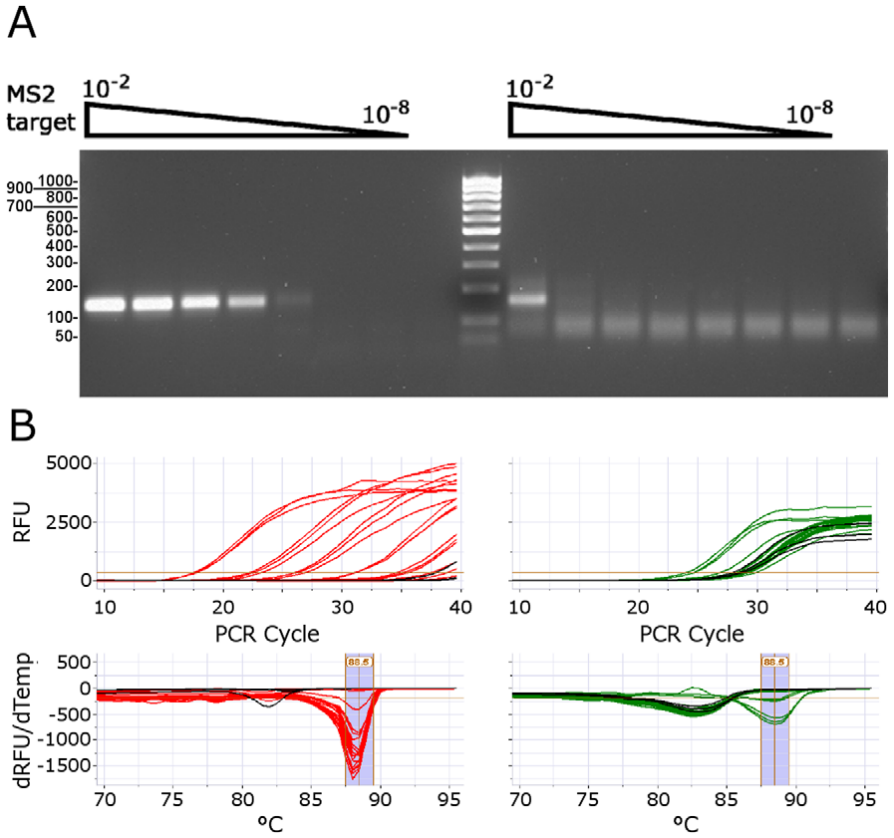
Comparison of 3173 Pol to Tth Pol in single enzyme RT-PCR detection of MS2 RNA. 3173 Pol, exonuclease minus (left, red) and *Tth* Pol (right, green) were used according to manufacturer recommendations to amplify a 10^2^ to 10^8^-fold dilution series of MS2 RNA and a water no target control using primers targeting the 160 bp product from Figure 6. **A.** Electrophoresis 2% agarose gel. Center lane is 100 bp DNA ladder. **B.** Top: Real-time PCR fluorescence in RFU (relative fluorescence units) vs. PCR cycles and Bottom: melt data in -dRFU/dTemperature vs. Temperature (°C). Blue region indicates melt curves for specific products.

The most common single enzyme RT-PCR method uses *Tth* Pol [23]. We compared the RT-PCR sensitivity, specificity and quantitation of the PyroScript mix with *Tth* Pol. The 160 bp MS2 target from Figure 6 was amplified by each enzyme over a dilution range of 10^−2^ to 10^−8^ (estimated at 120,000 to 0.12 copies) using manufacturer recommended conditions for each (Figure 7). The near single copy sensitivity and six-log linear detection range seen with PyroScript 3173 RT contrasts with the ∼120,000 copy detection limit and absence of a linear quantitation range seen with *Tth* Pol. A small amount of false product was detectable in the negative control by RT-qPCR but not by agarose gel. Significant false background PCR products generated by the *Tth* Pol system were readily detectable by both agarose gel and melt-curve analysis.

**Figure 8:**
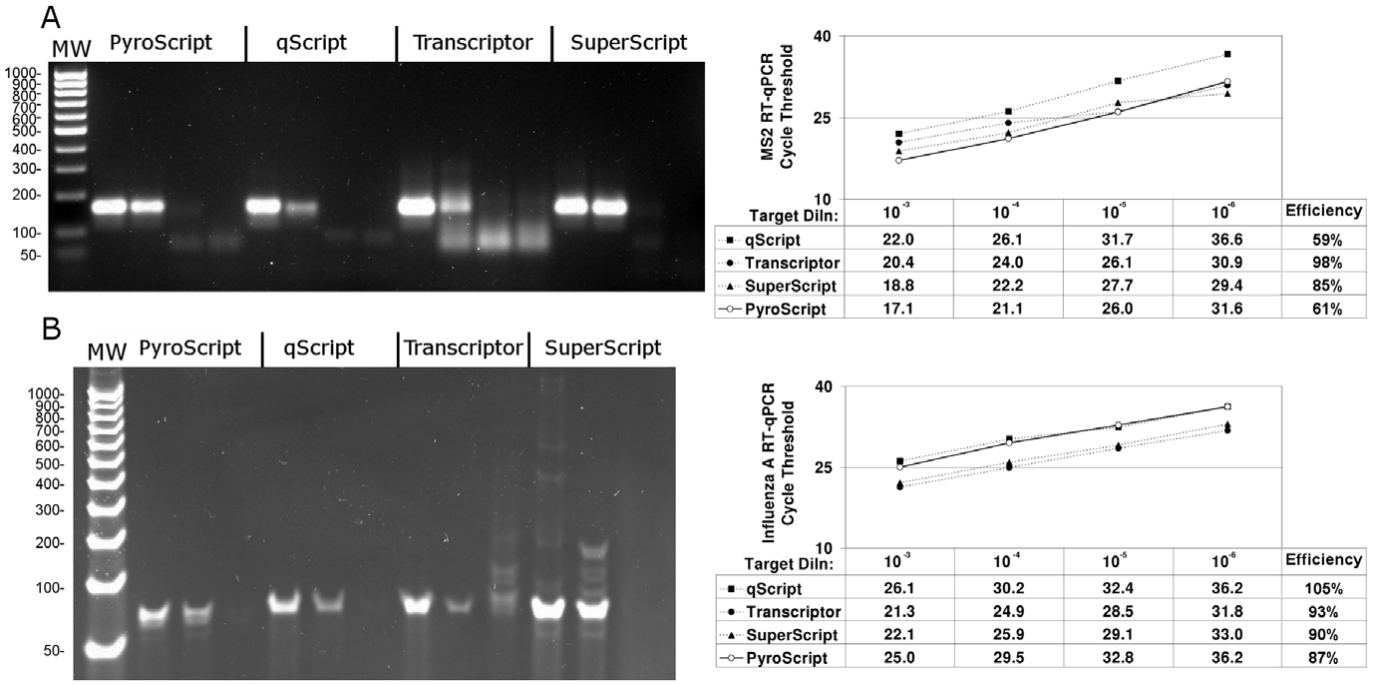
Comparison of 3173 Pol (PyroScript) RT-PCR mix with two enzyme RT-PCR systems in detection of MS2 and influenza A. Ten-fold serial dilutions of an MS2, an influenza A RNA preparation and a water only control (NTC) were amplified by one-step RT-PCR reagent mixes (PyroScript, qScript (Quanta), Transcriptor (Roche), and SuperScript (Invitrogen), as indicated. **A.** MS2 detection. Left panel: 2% agarose gel, each group of four wells are 10^−4^, 10^−6^, 10^−7^-fold target dilutions and NTC, MW is 100 bp DNA ladder (50 bp smallest band). Right Panel: RT-qPCR analysis of 10^−3^, 10^−4^, 10^−5^, and 10^−6^-fold target dilutions. **B.** Influenza A RNA detection. Left panel: 4–20% gradient polyacrylamide gel, each group of three wells are 10^−6^, 10^−7^-fold target dilutions and NTC, MW is 25 bp DNA ladder (50 bp smallest band). Right Panel: RT-qPCR analysis of 10^−3^, 10^−4^, 10^−5^, and 10^−6^-fold target dilutions.

Since two-enzyme systems using MMLV RT derivatives and *Taq* Pol are far more commonly used than single-enzyme systems, we compared the performance of single-enzyme PyroScript mix to three widely used mixes that are based on the two enzyme MMLV RT plus Taq Pol combination, but are referred to as “one-step” systems. The comparators were: SuperScript® III One-Step RT-PCR System with Platinum® Taq DNA Polymerase (Life Technologies), the qScript™ One-Step SYBR® Green qRT-PCR Kit (Quanta), and the Transcriptor® One-Step RT-PCR Kit (Roche). MS2 RNA extract was amplified using primers targeting the 160 bp product from Figure 6. Three dilutions of MS2 RNA (the lower dilutions from Figure 6D) and a water-only control were amplified by 40 cycles of RT-qPCR using each of the respective reagents (Figure 8A). All of the reagents appeared to have similar limits of detection and amplified the expected product as seen by electrophoresis. All of the reagents produced a weak background amplification product of about 60 bp, from both the lowest RNA dilution and the water-only control. The Transcriptor kit reproducibly amplified more false product than did the other three. Similar slopes from plots of qPCR cycle threshold versus fold target dilution show that all four reagent mixes amplified the MS2 target with similar efficiency although the qScript reagent appeared to amplify the target a few cycles later than the other three mixes.

The ability of these four reagents to amplify human influenza A virus RNA was also compared. Cultured influenza A Puerto Rico/8/1934 (H1N1) RNA was extracted from cell medium and amplified by 40 cycles of one-step RT-PCR (Figure 8B). The results with influenza A were similar to those seen for the MS2 target. All four reagents appeared to have similar limits of detection for the influenza A RNA extract and amplified the intended 60 bp target from the second lowest dilution of RNA. The PyroScript RT-PCR reaction was largely free of extraneous bands. In contrast, the Transcriptor and the Superscript-based mixes produced spurious bands, primarily of a size greater than the expected amplicon size. The Transcriptor kit also produced false products in the negative control reaction. Again the slopes of plots of qPCR cycle threshold versus fold dilution show that all four reagent mixes amplified the MS2 target with similar efficiency. In contrast with MS2, both the qScript and the PyroScript reagent were found to amplify influenza A several cycles later than the SuperScript and Transcriptor master mixes did.

## Discussion

The 3173 Pol based PyroScript RT-PCR master mix represents a practical alternative to two-enzyme (e.g. MMLV RTs/Taq Pol) RT-PCR systems and provides both theoretical and demonstrated advantages. No truly viable substitute for the two-enzyme systems has been described previously. Among bacterial DNA Pols that can be induced to use RNA templates, only the *Tth* Pol is thermostable enough for PCR, but its performance in RT-PCR in general has not proven competitive with the two-enzyme mixes. Since the upper limit for eukaryotic life is around 62°C, it seems unlikely that retroviruses will ever provide RTs thermostable for single-enzyme RT-PCR.

While the two-enzyme systems are widely used and generally reliable, deficiencies inherent in these systems have restricted certain improvements in RT-PCR. For example, secondary activities, including RNase H and terminal transferase, are associated with strand switching [4], [6] and insertion errors [42]. Replication of native retroviral genomes depends on specific sequences within the terminal repeats [43], which may be related to a significant bias seen with certain combinations of primer sequences and reverse transcriptases [44] used *in vitro*. In particular, the two 3′-terminal nucleotides of the primers can account for a 35,000-fold range in the frequency of misincorporation, a measured Km variation of 100-fold and Vmax range of several-fold when used with MMLV or AMV RTs [5]. These preferences are possible causes of amplification errors, amplification bias [8], poor concordance between tests [9], [45] and sequences that are completely refractory to reverse transcription [44], [45].

Extensive effort has been directed at engineering retroviral RTs to disable or eliminate the RNase H domain implicated in RT-dependent rearrangement [21]. Although such RTs produce fewer rearrangements, inactivation of RNase H also increases misincorporation and bias due to impaired amplification of specific sequences [3], [4], [46]. Additional mutations incorporated into SuperScript III RT (Life Technologies) to increase thermostability may have resulted in lower sensitivity [10] and exacerbated the interference with *Taq* Pol [16], but still have not provided adequate thermostability for single-enzyme RT-PCR. Alternative approaches of evolving or engineering thermostable Pols to use RNA templates [47], [48] have shown promise, but, to our knowledge, have not yet provided a commercial RT-PCR reagent.

To discover new thermostable enzyme activities, we investigated the previously unexplored resource encoded in the genomes of viral populations in thermal springs. Viruses are a highly abundant and diverse source of genetic variation [2], [49] and a promising source of new reagent enzymes [1]. A viral metagenomic library originating from a thermal hot spring provided a new enzyme, 3173 Pol, with efficient reverse transcription activity and thermostability for PCR. The physiological role of the RT activity of 3173 Pol is not clear. Lacking a cultivated virus/host combination, the replication mechanism of the source virus can only be inferred from sequence data. Based on the method of library construction, the virion has a double-stranded DNA genome. Thus, the overall viral replication mechanism is distinct from retroviruses.

In our experiments, the half-life of 3173 Pol at 94°C was 11 minutes compared to more than two hours for Taq Pol when assayed under the same conditions. A previously reported half-life of Taq Pol at 95°C is 20 minutes [50]. Although the thermostability of 3173 Pol is significantly lower than Taq and most other commonly used thermostable Pols, it is clearly adequate for PCR since product continues to accumulate up to forty cycles (Figures 6 and 7). The combination of thermostability and reverse transcriptase activity in one enzyme has practical implications. Because the two enzyme RT systems contain a thermolabile protein component, the use of hot start technologies to improve specificity of reverse transcription is not practical. The 3173 Pol should allow “hot start” methods to function during reverse transcription as well as amplification, which should improve specificity (data not shown).

The thermal profile of 3173 Pol (Figure 2) shows a peak of activity at 77°C, similar to Taq Pol, but nearly half of its activity remains at 55°C, significantly higher than the 10–20% reported for Taq Pol [50]. The higher reverse transcription temperature, combined with the strand displacement activity, should improve specificity and allow synthesis through difficult, structured and G/C rich RNA templates and may have been the basis of the lower amount of spurious products in PyroScript reactions seen in Figure 8. This broad thermal profile, strand displacement and initiation at nicks have enabled certain isothermal amplification schemes [51], [52] (manuscript in preparation).

One advantage of a thermostable RT is that the initial lower temperature incubation step can be eliminated, reducing overall reaction time and potentially increasing priming specificity. The high stability of 3173 Pol in solution compared to MMLV RT also allows the formulation of a complete PyroScript RT-PCR master mix, which lacks only analyte-specific primers and target. This formulation is stable for over a year at −20°C and simplifies reaction set up and reduces the potential for formulation errors.

A drawback of the two-enzyme systems is reduced efficiency during early rounds of RT-PCR amplification of low abundance targets when Taq Pol is used with MMLV RT [7], [12], [13], [14], [15], [16]. This inhibition has some sequence specificity [12], [16], which presumably biases amplification and may compromise the measurement of differential gene expression levels and the reliability of internal and external quantification standards. One explanation for this effect is that heating eliminates RT activity without fully disrupting DNA binding and this interferes with the efficiency of PCR amplification. The result is an underestimation of low abundance target concentration. If this is true, the availability of a single enzyme that reverse transcribes and amplifies should eliminate this effect.

We report other biochemical attributes likely to affect RT-PCR. Affinity for template influences the sensitivity and specificity of amplification, but has not been widely described for other Pols. This affinity can have an important impact on certain applications. For example, Bst Pol has a higher template affinity for DNA than Taq Pol, allowing use of lower template concentrations when DNA sequencing [53]. Also important is affinity for nucleotides. The nucleotide dissociation constants for Pol I enzymes from *T. thermophilus* and three thermostable *Bacillus* species were reportedly between 115 and 85 µM [54]. Processivity is probably related to affinity for template. The phi29 Pol has a processivity value of greater than 70,000 nt [27]. The processivity of 3173 (47 nt) is comparatively modest but still higher than either Bst or Taq Pols (37 and 9 nt, respectively). While processivity measurements are highly dependent on reaction conditions, the measured result for Taq is comparable with previously published values [50].

Although it is not as important for detection and quantification applications, fidelity is critical for preparative cDNA synthesis methods and for transcriptome sequencing. Published methods of fidelity measurement use DNA templates [35]. Using a variant of these methods, the wild-type 3173 Pol had a fidelity of 6.7×10^4^ similar to our measurements for the most accurate PCR enzymes, Pfu and Phusion Pols (5.8×10^4^ and 7.5×10^4^ respectively) when assayed in parallel. An exonuclease deficient mutant of 3173 Pol had a PCR fidelity of 0.9×10^4^, similar to the value measured for Taq Pol (1.4×10^4^) and slightly below the reported range of 2.5 to 5.0×10^4^ for Taq Pol [32], [35]. Published *in vitro* fidelity measurements for MMLV RT are especially difficult to compare since the assay conditions and temperatures are quite different; however, the reported fidelity for MMLV RT is between 1.7 and 3.0×10^4^ [55]. Measurement of the fidelity of 3173 Pol on RNA templates will require extensive studies beyond the scope of this report. If the fidelity on RNA is similar to the fidelity on DNA, 3173 Pol could prove especially valuable as an RNA sequencing enzyme for transcriptomics research. Thus, the determination of 3173 Pol fidelity is the basis of ongoing study.

The PyroScript mix was comparable in sensitivity to three leading commercial two-step RT-PCR kits when used to detect either MS2 phage or influenza A RNA. Background amplification in the absence of target, especially after 40 cycles of PCR, is problematic in clinical diagnostic tests where RNA target copies may approach single molecule levels. This problem is exacerbated in two-enzyme, one-step RT-PCR since the retroviral RTs are not thermostable and background reduction during reverse transcription using hot start methods for these RTs are not possible. We found that both the MS2 and the influenza one-step RT-PCR amplification reactions exhibited some propensity for non-specific product formation. However, all of the one-step kits produced background at similar or higher levels. Furthermore, these background products were often generated in earlier cycles than the 3173 enzyme false products. The yield of end product formed by the Transcriptor and by the Superscript reagents appeared greater than those of the Quanta and the PyroScript mixes (Figure 5), likely due to higher recommended amounts of primers used by these reactions (500 nM vs. 200 nM). However, the higher concentration of primers probably resulted in increased background observed with the Transcriptor and SuperScript reagents. With additional effort, reaction parameters for any of the enzymes could undoubtedly be optimized for specificity or for yield. This higher yield of end product does not appear to affect qPCR results.

While the PyroScript enzyme mix shows utility for a range of detection applications, we noted some limitations. The use of 3173 Pol for amplification of targets greater than about 350 nt is not reproducible although both the 3173 wild-type and exo- mutants generate PCR products from DNA targets up to 5 kb and higher (data not shown). This is consistent with the shorter length of cDNA products that we observed in the labeled primer extension experiments (Figure 3B). Figure 3C indicates a small amount of full-length, 714 nt cDNA product, although the bulk of product is less than 300 nt. Each of these shorter products terminates within a region of secondary structure of the MS2 RNA associated with RNase sensitivity [56] so these apparent size limits may reflect labile sites in target RNA and may have to do with RNA stability at the high extension temperatures used with 3173 Pol (72°C) than with inherent properties of the enzyme. Most detection modes amplify much shorter targets, but preparative RT-PCR with 3173 Pol will likely be affected by this observed limitation. In contrast to the two-enzyme mixes, use of 3173 Pol in RT-PCR was significantly more reliable when the primers were designed to anneal at the higher (72°C) annealing/extension temperature of the two step PCR protocol. Throughout the RT-qPCR studies, we used dye binding as the detection mode. An alternative detection chemistry uses hydrolysis probes commonly known as TaqMan® probes (Life Technologies) [57]. This chemistry was not tested since the 3173 Pol lacks the 5′-3′ exonuclease activity required to cleave a TaqMan probe. Finally, while the 3173 Pol reliably detected high abundance transcripts, as shown in Figure 6, it was noticeably less consistent with less abundant targets. The reason for this inconsistency is not fully understood and is under investigation. One explanation may be that the enzyme is sensitive to high abundances of non-target sequences, typical in total RNA extracts. This effect has been seen to a lesser extent with MMLV RT-based RT-PCR [10] and with optimization may be ameliorated. Such abundant non-target RNA is generally absent in viral RNA preparations and the 3173 Pol has proven especially useful for detection and quantification of RNA viruses. For detection of RNA viruses the PyroScript mix appears to be competitive with two enzymes systems that use a retroviral RT and a thermostable Taq Pol. Since RNA viruses including influenza, HIV, Dengue, West Nile and SARS coronavirus represent a substantial portion of emerging pathogens worldwide, an improved means of detecting and quantifying these viruses could have an important impact on global health care.

## Supporting Information

Table S1 Exonuclease Assay. The indicated number of units of enzyme were incubated with [33P]-labeled PCR product for 10 minutes at 70°C as described in methods. Shown are the percent counts released with background (water-only control) subtracted. Not detected is indicated when counts are not significantly above background counts, i.e. <10%.
(DOC)
