# Supplementary material for: Thermostable DNA Polymerase from a Viral Metagenome Is a Potent RT-PCR Enzyme
Source: PLoS One. 2012 Jun 4;7(6):e38371. doi: 10.1371/journal.pone.0038371 (PMC3366922; doi:10.1371/journal.pone.0038371)
Supplement: Table S1 — Exonuclease Assay. The indicated number of units of enzyme were incubated with [33P]-labeled PCR product for 10 minutes at 70°C as described in methods. Shown are the percent counts released with background (water-only control) subtracted. Not detected is indicated when counts are not significantly above background counts, i.e. <10%. (DOC) [file pone.0038371.s001.doc]

**Table S1. Exonuclease Assay**.

|  | % counts released | | | |
| --- | --- | --- | --- | --- |
| units | 3173 wt | Vent | Pfu | 3173 exo- |
| 0.01 | 0.20 | 0.21 | 0.05 | not detected |
| 0.05 | 0.27 | 0.52 | 0.20 | not detected |
| 0.1 | 0.34 | 1.38 | 0.09 | not detected |
| 0.5 | 2.06 | 2.61 | 0.28 | not detected |
| 2 | 13.46 | 21.31 | 3.14 | not detected |
| 5 |  |  |  | not detected |
| 100 |  |  |  | not detected |
